# APOL4, a Novel Immune-Related Prognostic Biomarker for Glioma

**DOI:** 10.3390/jcm11195765

**Published:** 2022-09-29

**Authors:** Hua Zhu, Xinyao Hu, Shi Feng, Yuntao Li, Yonggang Zhang, Sheng Qiu, Ran Chen, Yingze Ye, Lijuan Gu, Zhihong Jian, Ximing Xu, Xiaoxing Xiong

**Affiliations:** 1 Department of Neurosurgery, The Affiliated Huzhou Hospital, Zhejiang University School of Medicine, Huzhou Central Hospital, Huzhou 313000, China; 2 Department of Neurosurgery, Renmin Hospital of Wuhan University, Wuhan 430060, China; 3 Cancer Center, Renmin Hospital of Wuhan University, Wuhan 430060, China; 4 Central Laboratory, Renmin Hospital of Wuhan University, Wuhan 430060, China

**Keywords:** APOL4, glioma, prognosis, ICI, tumor-infiltrating cells

## Abstract

Glioma is the common, most aggressive and poorest prognostic tumor type in the brain. More and more biomarkers associated with glioma treatment, prognosis, and immunity are being discovered. Here, we aimed to explore the underlying biological functions and prognostic predictive value of Apolipoprotein L4 (APOL4) in glioma. We downloaded the expression data of APOL4 and clinical information from several databases and used R software for preprocessing. The clinical significance of APOL4 in a glioma outcome was explored by the Cox regression analysis and Kaplan–Meier survival analysis. In addition, immune infiltrates and microenvironmental indicators were assessed by CIBERSORT and TIMER. GO and KEGG analyses were used to analyze the potential functions of APOL4 in gliomas. APOL4 expression was increased in glioma specimens compared to normal tissues and correlated dramatically with the WHO grade. A survival analysis showed a shorter overall survival (OS) in glioma patients with APOL4 overexpression, and a Cox regression analysis showed that APOL4 was an independent prognostic factor for the OS of glioma patients. GSEA, GO, and KEGG enrichment analyses showed remarkable enrichment in immune-related pathways. APOL4 expression was positively correlated with immune infiltration (including DC cells, neutrophils, CD8+ T cells, B cells, macrophages, CD4+ T cells, etc.) and microenvironmental parameters (including immune, stromal, and ESTIMATE scores) in gliomas. Glioma patients with a higher expression of APOL4 may be more sensitive to immune checkpoint inhibitors (ICI). In conclusion, these findings suggest that APOL4 is associated with the tumor grade and immune infiltrates; APOL4 may be a new and potential biomarker for therapeutic and prognostic evaluations that may further suggest the therapeutic efficacy of immunotherapy.

## 1. Introduction

A glioma is the most common primary tumor in the brain, accounting for over 70% of malignant tumors of the central nervous system (CNS) [1]. Gliomas include astrocytomas, oligodendrogliomas, glioblastoma multiforme (GBM), and mixed tumors. GBM is the most common and most malignant grade 4 gliomas, with a median survival time of 15 months [1,2,3]. A combination of surgical resection, radiotherapy, and chemotherapy is still the standard of care for GBM, but its outcomes remain unsatisfactory, and the prognosis for patients is poor [4]. It is therefore urgent to find more effective treatments and to develop new indicators to predict patients’ response to treatment and patients’ prognosis in order to provide individual guidance on treatment modalities.

Apolipoprotein L4 (APOL4), a member of the lipoprotein L family, encodes a protein that may exert an action in reverse cholesterol transport from peripheral cells to the liver, as well as in lipid transport and exchange throughout the body (https://www.ncbi.nlm.nih.gov/gene/) (accessed on 11 January 2022). The APOL gene family is thought to be innate immune genes that have evolved rapidly in the primate lineage, and their expression is upregulated by pro-inflammatory signals such as interferon, tumor necrosis factor alpha and viral mimics [5,6]. Previous study has illustrated that lipid metabolism is largely reprogrammed in cancer and that most types of cancer use lipids and cholesterol to meet their unlimited energy needs and the demands of increased membrane biosynthesis. APOL4 has been found to be a prognostic marker for breast cancer [7]. However, no information is available on the role of APOL4 in glioma.

Bidirectional communication between the tumor microenvironment (TME) and cells is crucial for normal tissue homeostasis and tumor growth, and tumor-infiltrating immune cells (TIICs) are essential components of TME [8]. Cancer cells evade the surveillance and clearance of immune system through a range of mechanisms, and it is only when immune cells fail to destroy precancerous cells that cancer develops and progresses, making immunotherapy a promising approach to cancer treatment [9]. Immunotherapy focuses on using immune cells both within and outside of TME to recognize and attack cancer cells specifically and can be combined with conventional therapies such as chemotherapy, radiotherapy, and surgery to provide better tumor suppression [10]. The most widely investigated immunotherapy is ICI therapy and has achieved good results in some clinical trials [11]. However, the presence of the brain barrier has made the development of various immunotherapies in gliomas difficult. It is therefore urgent to identify new prognostic biomarkers for immunotherapy treatment.

In this study, we downloaded APOL4-related genetic and clinical information from The Cancer Genome Atlas (TCGA), Gene Expression Omnibus (GEO), and Chinese Glioma Genome Atlas (CGGA). After data preprocessing, we performed correlation, survival, and Cox regression analyses to explore the potential functions of APOL4. Our goal is to verify a new diagnostic biomarker for gliomas that will assist in disease stratification and precision treatment.

## 2. Methods

### 2.1. Data Obtain and APOL4 Expression Analysis

We obtained the mRNA expression profile datasets containing the clinical information from the public databases CGGA (http://www.cgga.org.cn/) (accessed on 21 February 2022) and TCGA (https://cancergenome.nih.gov/) (accessed on 25 February 2022). The TCGA database divides gliomas into LGG (low grade glioma) and GBM. In TCGA, the three cohorts (glioma, LGG, and GBM) were named TCGA_ LGG_GBM, TCGA_LGG, and TCGA_GBM, respectively. We applied R software to preprocess the gene expression profiles, including background correction, normalization, and log2 transformation. To evaluate the expression of APOL4 in the tissues of gliomas and other tumors, we employed the TIMRE database (https://cistrome.shinyapps.io/timer/) (accessed on 13 May 2022) to compare the expression of APOL4 in tumor and corresponding normal tissues. Additionally, the GEPIA (http://gepia.cancer-pku.cn/index.html) (accessed on 21 June 2022) was also used to explore the APOL4 expression in LGG, GBM, and normal tissues. The expression of APOL4 in different World Health Organization (WHO) grades (WHO 2021) was also examined, the same as we did [9]. The APOL4 protein levels in glioma and normal tissues was assessed by the HPA website (https://www.proteinatlas.org) (accessed on 28 June 2022). Moreover, the location of the APOL4 protein was also explored by the HPA.

### 2.2. Analyze the Prognostic Value of APOL4 in Glioma

As we reported before [12], Kaplan–Meier was used to evaluate the correlation of APOL4 with the prognosis of LGG and GBM patients, and the Cox regression analysis was applied to verify whether APOL4 can be regarded as an independent prognostic factor.

### 2.3. Evaluation of the Immune Infiltration and the Microenvironment

Using the “ESTIMATE” R package, immune and stromal cell communities were analyzed based on gene expression characteristics, and immune, stromal, and ESTIMATE scores were obtained [13]. We then used the CIBERSORT algorithm and TIMER database to evaluate the relationship between the APOL4 gene and the levels of different immune cells [14].

### 2.4. Assessment of Immune Checkpoints and ICI

The expression of several immune checkpoints was assessed in the APOL4-high and APOL4-low (The median was used to distinguish between high and low groups.) groups, as we previously studied [15]. The correlation of APOL4 with immune checkpoints was analyzed by Spearman’s correlation analysis. The TIDE scores and subclass mapping showing the sensitivity to ICI therapy were calculated in the APOL4-high and APOL4-low groups [16].

### 2.5. Potential Function Analysis of APOL4 in Glioma

The PPI network of APOL4 was conducted by STRING (https://cn.string-db.org/) (accessed on 1 July 2022) and GeneMANIA (https://cn.string-db.org/) (accessed on 18 June 2022). GSEA, GO, and KEGG analyses were used to explore the potential function of APOL4 in gliomas.

## 3. Results

### 3.1. APOL4 Was Upregulated in Glioma

**Figure 1:**
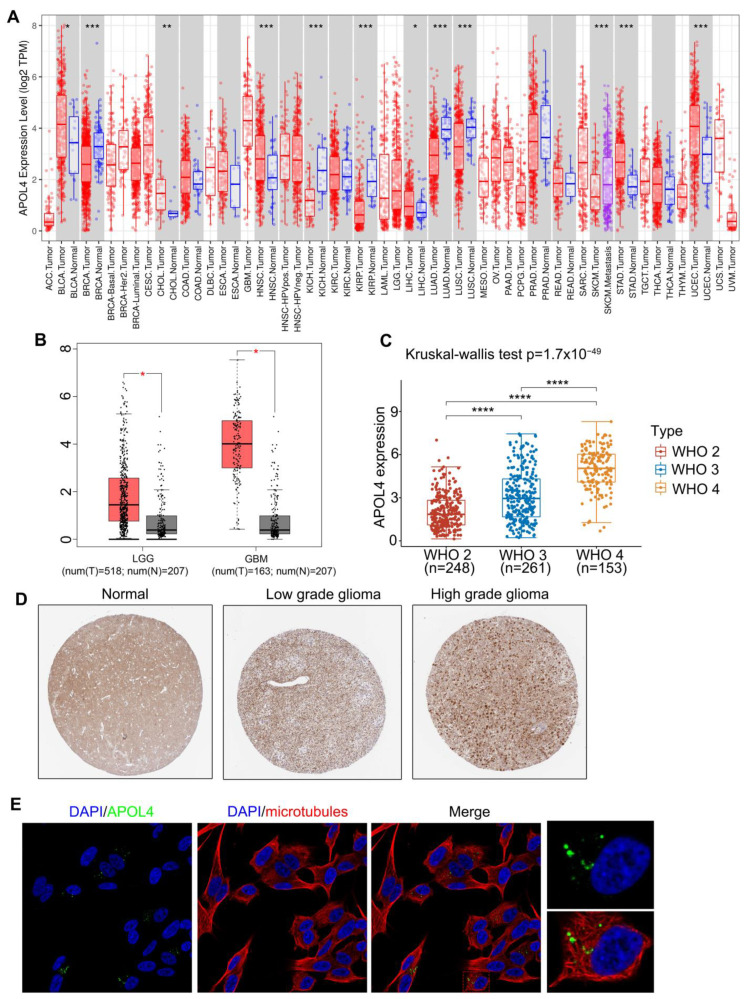
The expression of APOL4 in gliomas and other cancers. (**A**) The APOL4 expression in human tumors. (**B**) The APOL4 expression in LGG, GBM, and normal tissues. (**C**) The APOL4 expression in different WHO grades in gliomas. (**D**) The IHC staining of APOL4. (**E**) The immunofluorescence of APOL4 in the SH-SY5Y cell line. * *p* < 0.05, ** *p* < 0.01, *** *p* < 0.001, and **** *p* < 0.0001.

To explore the APOL4 expression in 33 human cancers (The full names and abbreviations are in Supplementary Materials), we employed the TIMER database, and we found that APOL4 was upregulated in ACC, CHOL, HNSC, LIHC, STAD, and UCEC but downregulated in BRCA, KIRP, LUAD, and LUSC compared with normal tissues (Figure 1A). To evaluate the expression of APOL4 in glioma and normal brain tissues, we further applied the GEPIA website, and the results showed that APOL4 mRNA was elevated in both LGG and GBM compared with normal brain tissues (Figure 1B). Moreover, APOL4 expression was related to the WHO grades, the expression of APOL4 in WHO 3 gliomas was higher than that in WHO grade 2 but lower than that in WHO grade 4 gliomas (*p* < 0.0001) (Figure 1C). Additionally, the protein level of APOL4 in gliomas was evaluated by the HPA database, and the IHC staining of APOL4 is shown in Figure 1D. The location of the APOL4 protein in the SH-SY5Y cell line (human neuroblastoma cell) was assessed, and the results indicated that APOL4 was located in the intracellular membrane (Figure 1E). These findings demonstrated that APOL4 was over-expressed in both LGG and GBM and was related to the WHO grades.

### 3.2. APOL4 Was an Independent Prognostic Factor

**Figure 2:**
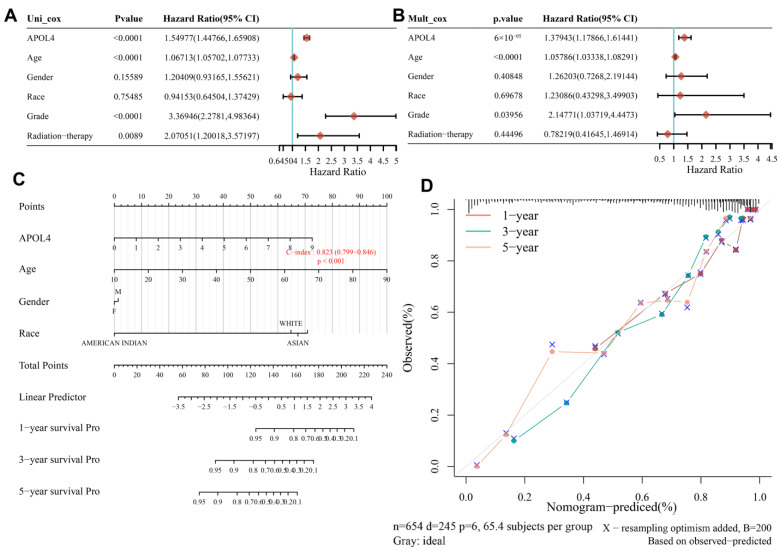
The prognostic value of APOL4. (**A**,**B**) Univariate and multivariate Cox regression. (**C**) Nomogram and calibration plots (**D**) predicting the OS of glioma patients.

**Figure 3:**
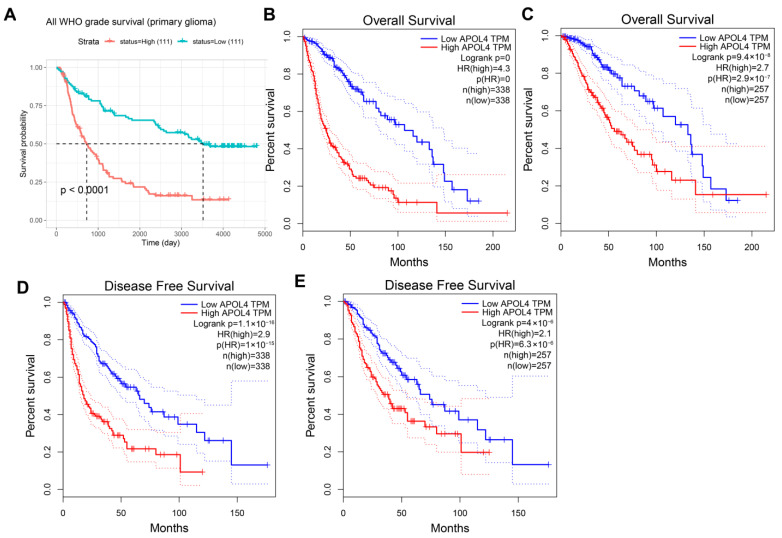
The APOL4 survival curves of gliomas. Compared to low expression, high APOL4 expression conferred a worse OS in glioma patients from the CGGA (**A**) and TCGA (**B**) databases. In the TCGA database, the association of APOL4 with OS (**C**) and DFS (**E**) in LGG. (**D**) The association of APOL4 with DFS in GBM.

To explore whether the overexpression of APOL4 can predict the prognosis of LGG and GBM. Univariate and multivariate Cox regression analyses were carried out, and the results indicated that APOL4 can be served as an independent prognostic factor (Figure 2A,B). The nomogram and the calibration curve demonstrated the ability of APOL4 to accurately predict patients 1, 3, and 5 prognoses (Figure 2C,D). Additionally, by analyzing the data in the CGGA database, we revealed that the high expression of APOL4 predicted a poorer prognosis in all WHO grade primary gliomas (Figure 3A). The data from TCGA demonstrated that the high expression of APOL4 was related to a poorer OS of gliomas (LGG + GBM) (*p* < 0.0001, Figure 2B) and LGG (*p* = 2.9 × 10^−7^, Figure 2C). The overexpression of APOL4 was also correlated with the poorer disease free survival (DFS) of gliomas (GLL + GBM) (*p* = 1 × 10^−15^, Figure 3D) and LGG (*p* = 6.3 × 10^−6^, Figure 3E). These findings indicated that the prognostic value of APOL4 in gliomas.

### 3.3. APOL4 Was Correlated with TIICs Infiltration in Glioma

**Figure 4:**
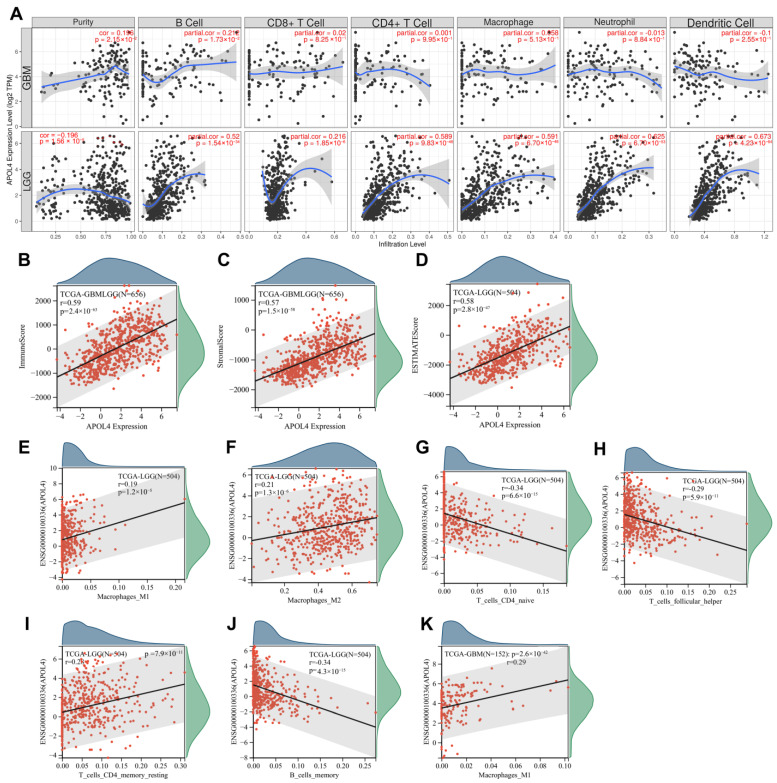
The connection between APOL4 and TIIC infiltration. (**A**) The relationship of APOL4 with TIIC infiltration in the TIMER database. The correlation between APOL4 and the immune (**B**), stromal (**C**), and ESTIMATE scores (**D**). The correlation of APOL4 with the infiltration of macrophages M1 (**E**), M2 (**F**), naïve CD4+ T cells (**G**), T cells follicular helper (**H**), T cells CD memory resting (**I**), and B cell memory (**J**) in LGG. (**K**) The relationship of APOL4 with the infiltration of macrophage M1 in GBM.

TIIC was an important component of TME and can influence the process and prognosis of tumors [17,18]. Herein, we explored the relationship between APOL4 and the TIIC infiltration levels. The results from the TIMER website indicated that APOL4 expression was associated with B-cell infiltrates in GBM (Cor = 0.212, *p* = 1.73 × 10^−2^) (Figure 4A). In LGG, APOL4 expression was associated with the infiltration of DCs (Cor = 0.673, *p* = 4.23 × 10^−64^), neutrophils (Cor = 0.625, *p* = 6.70 × 10^−53^), macrophages (Cor = 0.591, *p* = 6.70 × 10^−46^), CD4+ T cells (Cor = 0.589, *p* = 9.83 × 10^−46^), CD8+ T cells (Cor = 0.216, *p* = 1.85 × 10^−6^), and B cells (Cor = 0.52, *p* = 1.54 × 10^−34^) (Figure 4A). The APOL4 expression was highly positively correlated to the immune score (Cor = 0.59, *p* = 2.4 × 10^−63^) (Figure 4B), stromal score (Cor = 0.57, *p* = 1.5 × 10^−58^) (Figure 4C), and ESTIMATE score (Cor = 0.58, *p* = 2.8 × 10^−47^) (Figure 4D). Another algorithm, CIBERSORT, was further used to explore the correlation of APOL4 with TIIC infiltrates. The results indicated that APOL4 expression was positively associated with the infiltration of macrophage M1 (Cor = 0.19, *p* = 1.2 × 10^−5^) (Figure 4E), M2 (Cor = 0.21, *p* = 1.3 × 10^−6^) (Figure 4F), and resting CD4+ T cells (Cor = 0.28, *p* = 7.9 × 10^−11^) (Figure 4I) but negatively correlated with naïve CD4+ T cells (Cor = −0.34, *p* = 6.6 × 10^−15^) (Figure 4G) and memory B cells infiltration (Cor = −0.34, *p* = 4.3 × 10^−15^) (Figure 4J) in LGG. In GBM, AOPL4 expression was positively related to macrophage M1 (Cor = 0.29, *p* = 2.6 × 10^−4^) (Figure 4K). These findings indicated that APOL4 was associated with the immune milieu of gliomas.

### 3.4. APOL4 Was Correlated with the Response to ICI in Glioma Patients

**Figure 5:**
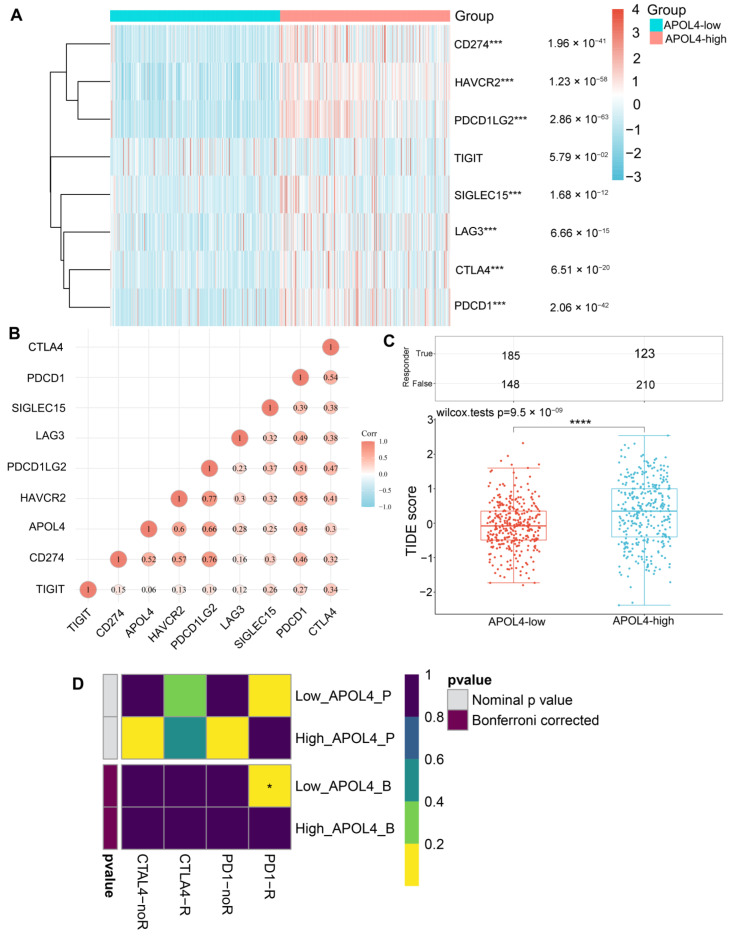
The relationship of APOL4 expression with immune checkpoints and the response to ICI in gliomas. (**A**) The expression of immune checkpoints in the APOL4-high and APOL4-low groups of gliomas. (**B**) The correlation of APOL4 with immune checkpoints. (**C**) TIDE scores in the APOL4-high and APOL4-low groups. (**D**) Submap analysis manifested that APOL4-low could be more sensitive to the PD1 inhibitor. * *p* < 0.05, *** *p* < 0.001, and **** *p* < 0.0001.

Immunotherapy, especially ICI, has revolutionized cancer treatments [19]. We further evaluated whether there was a correlation between APOL4 expression and the sensitivity of ICI therapy in glioma patients. We found that the several immune checkpoints, including CD274 (*p* = 1.96 × 10^−41^), CTLA4 (*p* = 6.51 × 10^−20^), HAVCR2 (*p* = 1.23 × 10^−58^), LAG3 (*p* = 6.66 × 10^−15^), PDCD1 (*p* = 2.06 × 10^−42^), PDCD1LG2 (*p* = 2.86 × 10^−62^), and SIGLEC15 (*p* = 1.68 × 10^−12^), were highly expressed in an APOL4-high group of gliomas (Figure 5A). The correlation of APOL4 with these immune checkpoints was further examined, and we found that APOL4 was positively related to several immune checkpoints, especially PDCDC1LG2 (Cor = 0.66), HAVCR2 (Cor = 0.6), CD274 (Cor = 0.52), and PDCD1 (Cor = 0.45) (Figure 5B). The Spearman correlation in GEPIA2.0 also verified these findings (Figure S1), and they indicated that APOL4 was positively associated with the expression of immune checkpoints. Moreover, we found that glioma patients who had a high expression of APOL4 had high TIDE scores (Figure 5C), which indicated that they may be more sensitive to ICI therapy. In addition to the TIDE prediction, we used subclass mapping to compare the two APOL4 expression subtypes with another published dataset (containing 47 melanoma patients who responded to immunotherapy). We found that APOL4-low is more promising to respond to anti-PD-1 therapy (Bonferroni corrected *p* < 0.05) (Figure 5D).

### 3.5. The Potential Role of APOL4 in Gliomas

**Figure 6:**
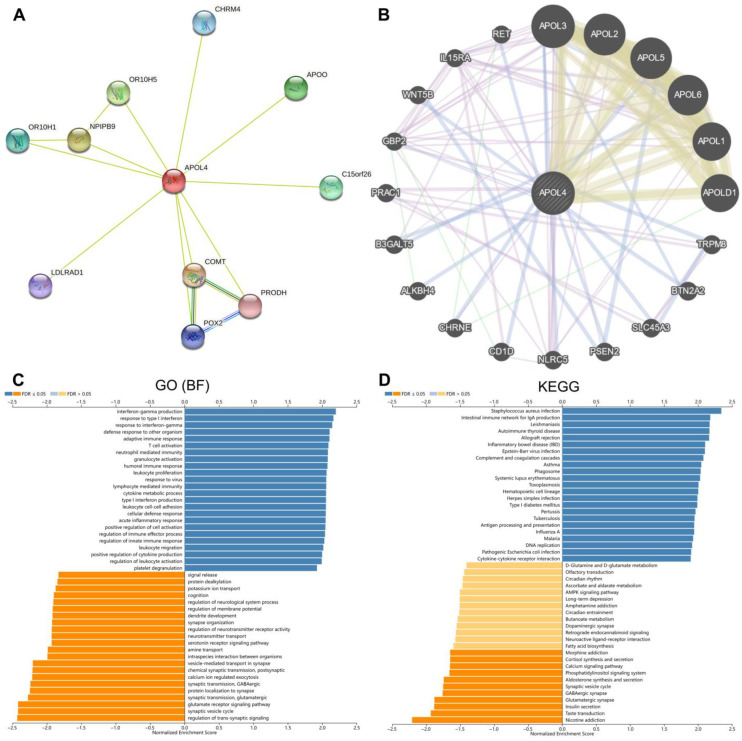
The potential role of APOL4 in gliomas. (**A**,**B**) The PPI network of APOL4 from the STRING and GeneMANIA databases, respectively. GO (**C**) and KEGG (**D**) analyses of APOL4 in gliomas.

**Figure 7:**
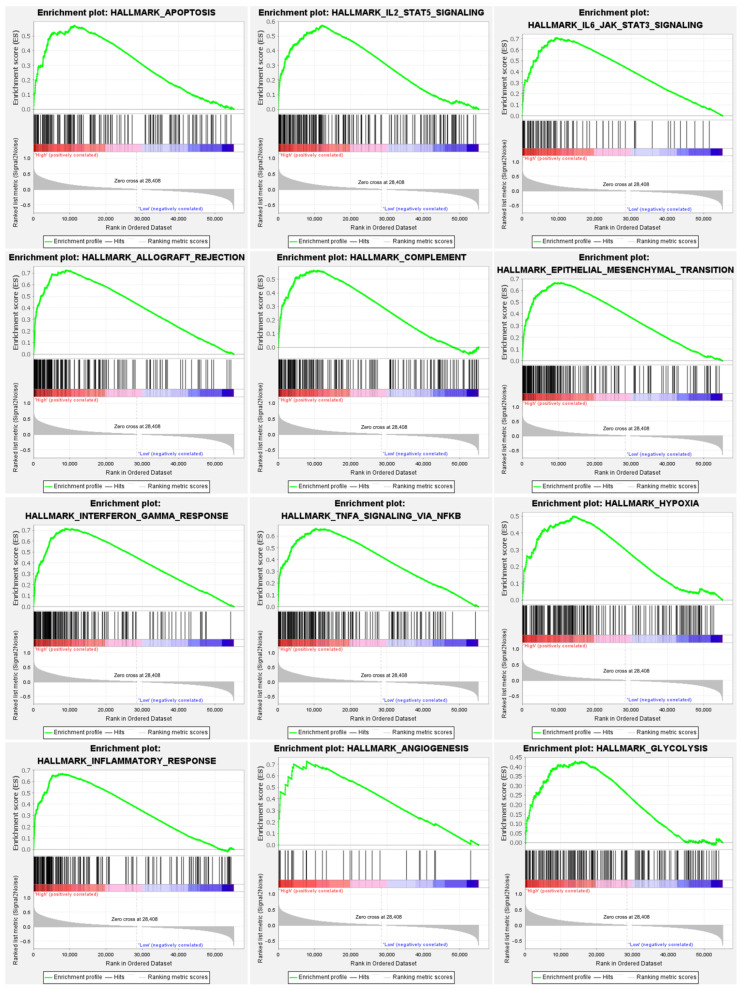
The GSEA analysis of APOL4 in gliomas.

To further discover the potential function of APOL4 in gliomas, we established the PPI network of APOL4 in the STRING and GeneMANIA webtools. The results from STRING indicated that APOL4 may interplay with APOO, CHRM4, C15orf26, COMT, PRODH, POX2, LALRAD1, OR10H1, NPIPB9, and OR10H5 (Figure 6A). The results in GeneMANIA indicated that APOL4 may interplay with APOL3, APOL2, APOL5, APOL6, APOL1, APOLD1, TRPM8, BTN2A2, SLC45A3, PSEN2, and NLRC5 (Figure 6B). The biological process the GO analysis demonstrated was that APOL4 was involved in numerous immune-related processes, including IFN-γ production, response to type I interferon, adaptive immune response, activation of T cells, neutrophil mediated immunity, granulocyte activation, humoral immune response, leukocyte proliferation, response to virus, lymphocyte mediated immunity, cytokine metabolic process, type I interferon production, leukocyte cell–cell adhesion, cellular defense response, regulation of innate immune response, positive regulation of immune effector process, leukocyte migration, positive regulation the production of cytokine, and regulation of leukocyte activation (Figure 6A). APOL4 was also related to the regulation of trans-synaptic signaling, synaptic vesicle cycle, and the glutamate receptor-signaling pathway in gliomas (Figure 6A). The KEGG pathway analysis illustrated that APOL4 was associated with the staphylococcus aureus infection, intestinal immune network of IgA production, leishmaniasis, autoimmune thyroid disease, allograft rejection, asthma, phagosome, systemic lupus erythematosus, toxoplasmosis, hematopoietic cell lineage, herpes simplex infection, type I diabetes mellitus, inflammatory bowel disease, pertussis, tuberculosis, antigen processing and presentation, DNA replication, and cytokine–cytokine receptor interaction in gliomas (Figure 6D). Additionally, APOL4 was related to nicotine addiction, taste transduction, and glutamatergic synapse in gliomas (Figure 6D). The GSEA analysis indicated that several immune-related pathways were positively correlated to high APOL4 expression, including apoptosis, IL2-STAT5 signaling, IL6-JAK-STAT3 signaling, allograft rejection, complement, epithelial–mesenchymal transition, interferon gamma response, TNFα signaling via NF-κB, hypoxia, inflammatory response, angiogenesis, and glycolysis (*p* < 0.05) (Figure 7). These findings indicated that APOL4 may participate in the immune-related pathways in gliomas.

## 4. Discussion

A glioma is a common primary malignant brain tumor in adults, and GBM is the most aggressive and incurable type, with a high recurrence rate and a high mortality rate [20]. The advent of immunotherapy and targeted therapies has brought new hope to patients with gliomas. A range of biomarkers have been identified in the local tumor tissue, but there is a lack of biomarkers that can be detected [21]. Therefore, our work has been devoted to finding suitable targets for predictive immunotherapy.

APOL4 is a member of the lipoprotein L family, which encodes a protein that may play a role in the reverse transport of cholesterol from peripheral cells to the liver and in the transport and exchange of lipids throughout the body. The APOL gene family is thought to be innate immune genes that evolved rapidly in the primate lineage, and their expression is upregulated by proinflammatory signals [5,6]. It has been reported that APOL4 may be a prognostic marker for breast cancer [7]. However, whether these is a relationship between APOL4 and glioma has not been studied.

In the current study, we found that APOL4 mRNA expression was elevated in gliomas and closely correlated with the tumor grade, with the highest expression in GBM and higher WHO grade 3 than WHO grade 2. APLO4 is highly expressed in GBM and may serve as a predictor. This suggests that APOL4 is a marker of poor prognostic outcomes. The analysis of the clinical survival data also confirmed that patients with gliomas with high APOL4 expression had significantly shorter survival times than those with low expression. Moreover, the Cox regression analysis confirmed that APOL4 was an independent prognostic biomarker for gliomas. These findings indicated that APOL4 may be engaged in the malignant biological process of gliomas, and its expression may be severed to predict the outcome of gliomas. However, it is not clear exactly how APOL4 mediates the development of gliomas.

By analyzing the association of APOL4 with TIICs in the tissues of LGG and GBM, we found that APOL4 was positively related to the immune, stromal, and ESTIMATE scores. In GBM, APOL4 was positively associated with the macrophages and B-cell infiltration. In LGG, APOL4 was positively correlated with several immune cells, especially DCs, neutrophils, macrophages, CD4+ T cells, and B cells. These findings illustrate that APOL4 may modulate the immune microenvironment of gliomas. However, the exact mechanism has not been clarified. Additionally, we found that the expression of several immune checkpoints was higher in the APOL4-high group than that in the APOL4-low group of gliomas. Moreover, we revealed a positive connection between APOL4 and CTLA4, PDCD1, PDCCD1LG2, HAVCR2, and CD274. The expression of these immune checkpoints indicated the poor prognosis of glioma patients. Tumor cells tend to evade cytotoxic T-lymphocyte destruction by upregulating immune checkpoints ligands, such as PD-L1, which can lead to the suppression of lymphocyte activation [12]. APOL4 may regulate the process of gliomas by interplaying or regulating immune checkpoints. The application of ICI can block the expression of immune checkpoints and can reactivate T cells and reshape the TME. However, it is the heterogeneity of the tumor, altered checkpoints, and extensive immunosuppression in the tumor microenvironment that complicates the treatment of gliomas. Therefore, it is of great importance to predict the response of patients to ICI therapy. Herein, we revealed that the expression of APOL4 may predict the response of glioma patients to ICI therapy. Glioma patients with a high expression of APOL4 may be more sensitive to ICI. These results illustrated that APOL4 may be regarded as a novel biomarker that can predict the response to ICI therapy in glioma patients. 

The biological process GO analysis demonstrated that APOL4 was involved in numerous immune-related processes, including IFN-γ production, response to type I interferon, adaptive immune response, T-cell activation, neutrophil mediated immunity, granulocyte activation, humoral immune response, leukocyte proliferation, response to virus, lymphocyte mediated immunity, cytokine metabolic process, type I interferon production, leukocyte cell–cell adhesion, cellular defense response, positive regulation of immune effector process, regulation of innate immune response, leukocyte migration, positive regulation of cytokine production, and the regulation of leukocyte activation. The KEGG pathway analysis illustrated that APOL4 was associated with antigen processing and presentation, DNA replication, cytokine–cytokine receptor interaction in glioma, revealing the potential functions of APOL4 in gliomas. The GSEA analysis indicated that several immune-related pathways were positively correlated to a high APOL4 expression, including apoptosis, IL2-STAT5 signaling, IL6-JAK-STAT3 signaling, allograft rejection and complement, epithelial–mesenchymal transition, interferon gamma response, TNFα signaling via NF-κB, hypoxia, inflammatory response, angiogenesis, and glycolysis. As previously reported, the IL6/JAK/STAT3 pathway was involved in the B7-H4(B7x)-mediated cross-talk between glioma-initiating cells and macrophages [22]. Additionally, angiogenesis and glycolysis also exert actions in the progress of tumors [23]. These findings further demonstrated that APOL4 was an immune-related biomarker in gliomas.

In conclusion, we found that APOL4 is aberrantly expressed in LGG and GBM than normal tissues, and high APOL4 expression predicts a poor outcome of glioma patients. This work also revealed that APOL4 may be a novel biomarker for the prognosis and ICI treatment of gliomas.

## Data Availability

The datasets presented in this study can be found in online repositories. The names of the repository/repositories and accession number(s) can be found in the article.

## Supplementary Materials

The following are available online at https://www.mdpi.com/article/10.3390/jcm11195765/s1: Figure S1: Correlation of APOL4 with immune checkpoints in GEPIA. Table S1: Full names and abbreviations of 33 types of cancer.
