# Supplementary material for: APOL4, a Novel Immune-Related Prognostic Biomarker for Glioma
Source: J Clin Med. 2022 Sep 29;11(19):5765. doi: 10.3390/jcm11195765 (PMC9572388; doi:10.3390/jcm11195765)
Supplement: Supplementary file 1 [file jcm-11-05765-s001.zip › supplementary table S1.pdf]

Table S1. Full names and abbreviations of 33 types of cancer.

Acute Myeloid Leukemia	(LAML)
Adrenocortical Cancer	(ACC)
Bile Duct Cancer	(CHOL)
Bladder Cancer	(BLCA)
Breast Cancer	(BRCA)
Cervical Cancer	(CESC)
Colon Cancer	(COAD)
Endometrioid Cancer	(UCEC)
Esophageal Cancer	(ESCA)
Glioblastoma	(GBM)
Head and Neck Cancer	(HNSC)
Kidney Chromophobe	(KICH)
Kidney Clear Cell Carcinoma	(KIRC)
Kidney Papillary Cell Carcinoma	(KIRP)
Large B-cell Lymphoma	(DLBC)
Liver Cancer	(LIHC)
Lower Grade Glioma	(LGG)
Lung Adenocarcinoma	(LUAD)
Lung Squamous Cell Carcinoma	(LUSC)
Melanoma	(SKCM)
Mesothelioma	(MESO)
Ocular melanomas	(UVM)
Ovarian Cancer	(OV)
Pancreatic Cancer	(PAAD)
Pheochromocytoma & Paraganglioma	(PCPG)
Prostate Cancer	(PRAD)
Rectal Cancer	(READ)
Sarcoma	(SARC)
Stomach Cancer	(STAD)
Testicular Cancer	(TGCT)
Thymoma	(THYM)
Thyroid Cancer	(THCA)
Uterine Carcinosarcoma	(UCS)
