# Supplementary figures and images for: APOL4, a Novel Immune-Related Prognostic Biomarker for Glioma
Source: J Clin Med. 2022 Sep 29;11(19):5765. doi: 10.3390/jcm11195765 (PMC9572388; doi:10.3390/jcm11195765)

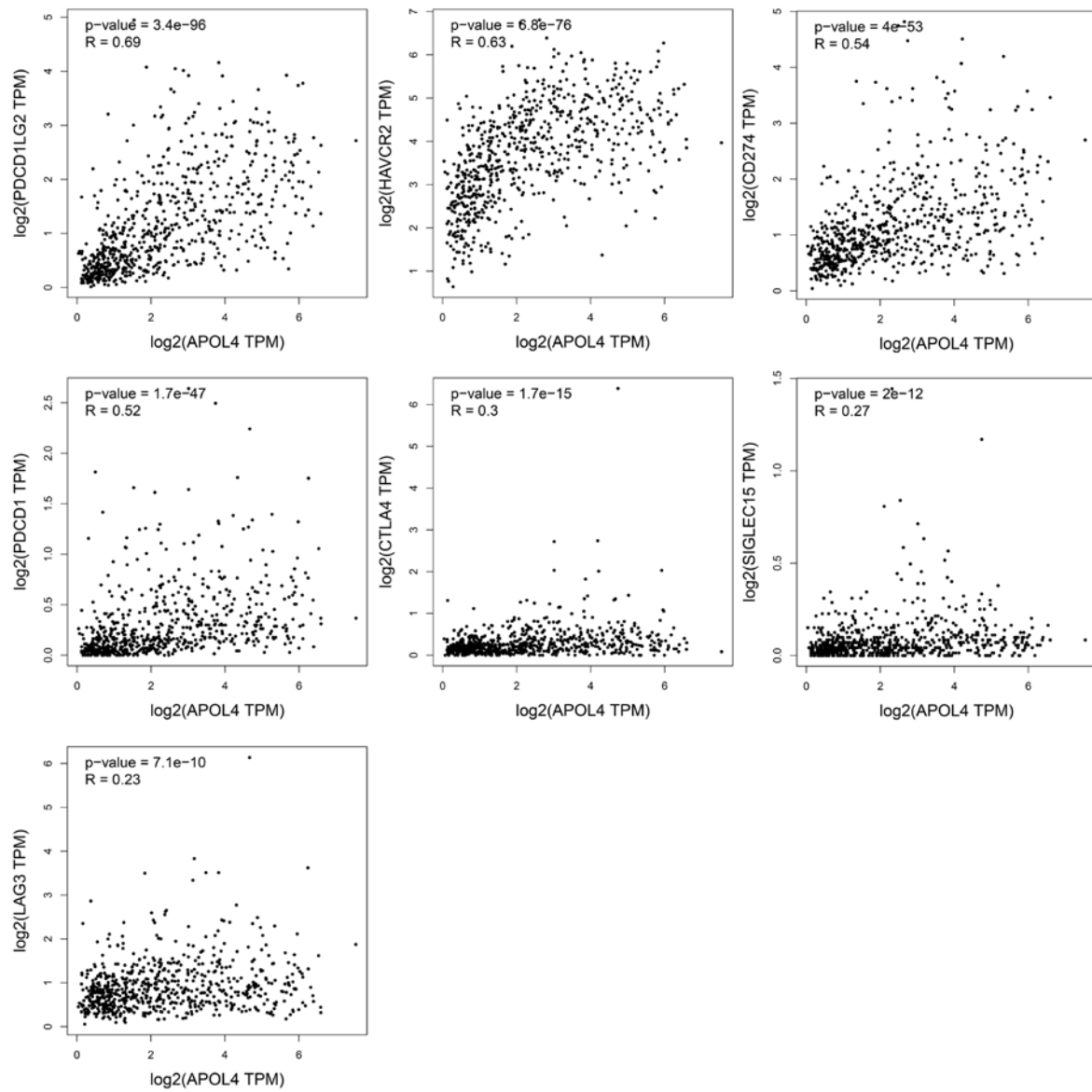

**Figure S1.** Correlation of APOL4 with immune checkpoints in GEPIA.

Supplement: Supplementary file 1 [file jcm-11-05765-s001.zip › Figure S1.pdf]
